# Screening of phospholipase A activity and its production by new actinomycete strains cultivated by solid-state fermentation

**DOI:** 10.7717/peerj.3524

**Published:** 2017-07-06

**Authors:** Priscila Sutto-Ortiz, María de los Angeles Camacho-Ruiz, Manuel R. Kirchmayr, Rosa María Camacho-Ruiz, Juan Carlos Mateos-Díaz, Alexandre Noiriel, Frédéric Carrière, Abdelkarim Abousalham, Jorge A. Rodríguez

**Affiliations:** 1 Biotecnología Industrial, Centro de Investigación y Asistencia en Tecnología y Diseño del Estado de Jalisco A.C., Zapopan, Jalisco, Mexico; 2 Univ Lyon, Université Lyon 1, Institut de Chimie et de Biochimie Moléculaires et Supramoléculaires (ICBMS), UMR 5246, Métabolisme, Enzymes et Mécanismes Moléculaires (MEM2), Villeurbanne Cedex, France; 3 CNRS, Aix Marseille Université, UMR 7282, Enzymologie Interfaciale et de Physiologie de la Lipolyse, Marseille, France; 4 Departamento de Fundamentos del Conocimiento, Centro Universitario del Norte, Universidad de Guadalajara, Colotlán, Jalisco, Mexico

**Keywords:** Phospholipase A, Phospholipids, Actinomycetes, High-throughput screening, Solid-state fermentation

## Abstract

Novel microbial phospholipases A (PLAs) can be found in actinomycetes which have been poorly explored as producers of this activity. To investigate microbial PLA production, efficient methods are necessary such as high-throughput screening (HTS) assays for direct search of PLAs in microbial cultures and cultivation conditions to promote this activity. About 200 strains isolated with selected media for actinomycetes and mostly belonging to *Streptomyces* (73%) and* Micromonospora* (10%) genus were first screened on agar-plates containing the fluorophore rhodamine 6G and egg yolk phosphatidylcholine (PC) to detect strains producing phospholipase activity. Then, a colorimetric HTS assay for general PLA activity detection (cHTS-PLA) using enriched PC (≈60%) as substrate and cresol red as indicator was developed and applied; this cHTS-PLA assay was validated with known PLAs. For the first time, actinomycete strains were cultivated by solid-state fermentation (SSF) using PC as inductor and sugar-cane bagasse as support to produce high PLA activity (from 207 to 2,591 mU/g of support). Phospholipase activity of the enzymatic extracts from SSF was determined using the implemented cHTS-PLA assay and the PC hydrolysis products obtained, were analyzed by TLC showing the presence of lyso-PC. Three actinomycete strains of the *Streptomyces* genus that stood out for high accumulation of lyso-PC, were selected and analyzed with the specific substrate 1,2-α-eleostearoyl-*sn*-glycero-3-phosphocholine (EEPC) in order to confirm the presence of PLA activity in their enzymatic extracts. Overall, the results obtained pave the way toward the HTS of PLA activity in crude microbial enzymatic extracts at a larger scale. The cHTS-PLA assay developed here can be also proposed as a routine assay for PLA activity determination during enzyme purification,directed evolution or mutagenesis approaches. In addition, the production of PLA activity by actinomycetes using SSF allow find and produce novel PLAs with potential applications in biotechnology.

## Introduction

Phospholipases A (PLAs) belong to a large family of lipolytic enzymes including PLA_1_ (EC 3.1.1.32) and PLA_2_ (EC 3.1.1.4) that catalyze preferentially the hydrolysis of the ester bond at position *sn*-1 and *sn*-2 of glycerophospholipids, respectively. PLAs play a central role in a wide variety of physiological processes generally including phospholipid digestion and metabolism, but some may also be involved in host defense against bacterial infections, remodeling of cell membrane, signal transduction (Dennis, 2015) and pathophysiological processes by producing precursors of various types of biologically active lipid mediators (Lambeau & Gelb, 2008; Murakami et al., 2014), having a role in virulence factor for invasion of host cells such as microbial PLAs (Ghannoum, 2000) or promoting cytotoxic effects such as PLAs found in venoms (Kini, 2005).

Nowadays, some PLAs are used in scientific and medical research, e.g., into the therapeutic field through inhibitors design for the creation of anti-inflammatory agents and as diagnostic markers for microbial infections (Karabina et al., 2010). Besides, PLAs have several industrial and biotechnological applications such as cosmetic preparations, vegetable oil refining by degumming (Clausen, 2001), production of modified phospholipids, bread making, dairy products, modification of egg yolk (Campbell, Te Bokkel & Thatcher, 2004), starch industry, animal feed additives, synthesis of surfactants and detergents (Ramrakhiani & Chand, 2011). However, although the use of phospholipases for industrial purposes is considered environmentally friendly and cost-effective, there is a necessity for the discovery of novel enzymes that might be more beneficial for biotechnological uses (Borrelli & Trono, 2015).

This steadily increased industrial demand of original and efficient phospholipases has stimulated the search of novel natural sources. In particular, PLAs are widespread in nature and have been found in various organisms like bacteria, fungi, protozoa, mammals, invertebrates and plants (Ramrakhiani & Chand, 2011). Within them, microbial phospholipases are of major interest due to the fact that their gene cloning and expression in microbial hosts is relatively easy, they can be improved trough protein engineering (Song, Han & Rhee, 2005) and the number of solved crystal structures is in constant increase (Matoba, Katsube & Sugiyama, 2002; Murayama et al., 2013).

Novel microbial phospholipases can be presumably found in actinomycetes, which are filamentous Gram-positive bacteria that exhibit true branching like fungi. These microorganisms are widely distributed in terrestrial environments and have long been an interesting source of other commercially valuable enzymes and therapeutically useful bioactive molecules (Clausen, 2001; Cook & Meyers, 2003; Lazzarini et al., 2000; Matoba, Katsube & Sugiyama, 2002).

Actinomycetes can be cultivated by submerged fermentation (SmF) or solid-state fermentation (SSF). SmF has been used widely for bioactive compound production from actinomycetes, such as Lipstatin (an inhibitor of pancreatic lipases) by *Streptomyces toxytricini* (Weibel et al., 1987), bonactin (that displayed antimicrobial activity against both Gram-positive and Gram-negative bacteria as well as antifungal activity) by the marine *Streptomyces* sp. BD21-2 (Schumacher et al., 2003), diazepinomicin (with antibacterial, anti-inflammatory and antitumor activity) by a marine *Micromonospora* strain (Manivasagan et al., 2014), and enzyme production such as L-Asparaginase by *Streptomyces ginsengisoli* (Deshpande, Choubey & Agashe, 2014). With less use, SSF has been employed to produce antibiotics such as meroparamycin (El-Naggar, El-Assar & Abdul-Gawad, 2009), and enzymes such as lipases from marine actinomycetes (Mary Suji, Priya & Sivaraj, 2014).

SSF has emerged as a potential technology for enzyme production at industrial scale that could employ solid material as either a natural substrate or an inert support (Holker, Hofer & Lenz, 2004; Pandey et al., 1999). On a gram bench-scale, SSF appears to be superior to the traditional SmF technique in several aspects such as higher fermentation productivity, higher end-concentration of products, higher product stability, lower catabolic repression, cultivation of microorganisms specialized for water-insoluble substrates, lower demand on sterility due to the low water activity, lower energy requirement and less wastewater production with lesser risk of bacterial contamination (Viniegra-Gonzalez et al., 2003; Hansen et al., 2015; Holker, Hofer & Lenz, 2004; Thomas, Larroche & Pandey, 2013). SSF is particularly attractive because it could provide an economical and environmentally friendly production (Hansen et al., 2015; Thomas, Larroche & Pandey, 2013) of microbial products such as biofuel, food, industrial chemicals and pharmaceutical products (Chen & He, 2012). Particularly, SSF holds tremendous potential for enzyme production like lipases (Mateos-Diaz et al., 2006), cellulases (Hansen et al., 2015), tannases (Aguilar et al., 2001), or production of metabolites (Hesseltine, 1972; Lakshminarayana et al., 1975), mainly by fungal species. Because of these particularities, and the low inhibition by substrate (Diaz-Godinez et al., 2001; Holker, Hofer & Lenz, 2004) and because of the advantage to use more quantity of inductors such as lipids (Damaso et al., 2008; Kumar & Ray, 2014) than SmF (Acikel, Ersan & Sag Acikel, 2011), SSF may offer an opportunity to produce phospholipase activity from actinomycetes.

Besides the microbial source of phospholipase activity, sensitive and reliable high-throughput screening (HTS) assays should be devised to detect PLA activity. For a general detection of PLA activity, several time-consuming or expensive methods have been proposed using chromogenic (Lobo de Araujo & Radvanyi, 1987; Petrovic et al., 2001; Price, 2007) or fluorogenic (Feng et al., 2002) substrates. However, a rapid and reliable method for HTS is still a challenge for efficient detection of potential phospholipases. In this sense, a ultraviolet (UV) spectrophotometric assay for HTS of lipases was recently developed by our group, based on the use of triacylglycerols (TAG) with UV absorption obtained from *Aleurites fordii* seeds oil (or tung oil) (Mendoza et al., 2012; Serveau-Avesque et al., 2013) that contains up to 70% of α-eleostearic acid (9*Z*, 11*E*, 13*E*-octadecatrienoic acid) (Pencreac’h et al., 2002). Based on the same principle, subsequently it was developed the specific phospholipid 1,2-α-eleostearoyl-*sn*-glycero-3-phosphocholine (EEPC), similar to that reported by (Wolf, Sagaert & Bereziat, 1981) and esterified at position *sn*-1 and *sn*-2 with α-eleostearic acid for specific detection of PLA activity (El Alaoui et al., 2014). Nevertheless, these substrates with UV absorption properties are not commercially available, and have not yet been applied into a real screening study with microbial extracts. More recently, a HTS method for lipases/esterases was reported (Mateos-Diaz et al., 2012), using partially soluble (tributyrin) or insoluble (trioctanoin) triglycerides substrates, *p*-nitrophenol as a pH indicator, Triton^®^ X-100, CHAPS or N-lauroylsarcosine as emulsifiers and β-cyclodextrin as a fatty acid captor. This method allows a quantitative detection of the enzymatic activity proving to be useful for screening a high number of carboxylester hydrolases from wild isolates or variants generated by directed evolution. Afterwards, a variant of this method was proposed for the screening of acidic, neutral, or alkaline lipases using the same substrates but with a variety of pH indicators like bromocresol green, chlorophenol red, cresol red and thymol blue (Camacho-Ruiz et al., 2015). This method known as pH indicator-based lipase assay (PHIBLA) is compatible with a high sample throughput and can be applied for the screening of lipases and lipase inhibitors from biological samples. However, these methods are not adapted to efficiently determine and screening PLA activity.

Considering these statements, the purpose of this work was to search and produce PLA enzymes from actinomycetes, envisioning potential applications in biotechnology. On the one hand, it was necessary to implement a set of techniques that allow an efficient search of PLA activity within a large number of microorganisms. To achieve this objective, an agar-plate method and a colorimetric HTS assay for general PLA activity detection (cHTS-PLA) were developed using enriched phosphatidylcholine (egg PC ≈ 60%) as substrate combined with fluorophore and pH-indicators, respectively. These methods were applied on various microbial collections from Mexican environments. On the other hand, the production of PLA by microorganisms was implemented using SSF with the agricultural by-product sugar-cane bagasse and phospholipid as inductor. Finally, the phospholipase activity type A in enzymatic extracts was confirmed by using TLC analysis and the specific substrate EEPC. To our knowledge, this is the first time that the screening of PLA activity in actinomycete strains and production of these enzymes by SSF is studied.

## Materials and Methods

### Reagents and media

Gum arabic, bacteriological agar, β-cyclodextrin (β-CD), casein, cresol red, L-α-phosphatidylcholine from egg yolk ≈60% TLC (PC), N-lauroylsarcosine (NLS), butylhydroxytoluene (BHT), oleic acid, rhodamine 6G, sodium taurodeoxycholate (NaTDC), 3-(N-morpholino)propanesulfonic acid (MOPS), ethylenediaminetetraacetic acid (EDTA), Tween 80, porcine pancreatic PLA_2_ (ppPLA_2_), PLA_1_ from *Thermomyces lanuginosus* (tlPLA_1_) and honey bee venom (*Apis mellifera*) PLA_2_ (hbPLA_2_) were purchased from Sigma-Aldrich-Fluka (St. Louis, MO, USA). Recombinant human pancreatic lipase (rHPL) was expressed in *Pichia pastoris* and purified from culture media as described by Belle et al. (2007). Recombinant guinea pig pancreatic lipase-related protein 2 (rGPLRP2) was expressed in *Aspergillus oryzae* and purified according to Hjorth et al. (1993). Yeast and malt extracts were purchased from BD (Denver, CO, USA). 1,2-α-eleostearoyl-*sn*-glycero-3-phosphocholine (EEPC) was synthesized as described by El Alaoui et al. (2014). Thin-layer chromatography (TLC) aluminum sheets, coated with 0.2 mm silica gel 60 F_254_, were purchased from Merck. Sugar-cane bagasse support was kindly donated by the sugar refinery San Francisco Ameca S.A. de C.V. (Ameca, Jalisco, México). All other reagents were obtained from local suppliers.

### Microbial collections

Microorganisms analyzed in this investigation were isolated from different Mexican environments. Collection 1 was obtained from lake sediments (1.5 m depth) of regions of Lake Chapala located in Michoacán (Bahía de Cojumatlán) and Jalisco (San Luis Soyatlán, Tuxcueca and San Juan Cosalá), states of Mexico where ambient temperature ranging from 10 to 35°C. Samples from Collection 2 were obtained from uncultivated soil of a wet region within the area called Las Tortugas located in Jalisco with temperatures from 16 to 24°C. Microorganisms from Collection 3 were kindly donated by Dr. Ali Asaff Torres (CIAD, Sonora, México) and obtained from soil and dry crusts of the Sonoran desert, a region in the north of Mexico with extreme temperatures ranging from 0 to 50°C. Strains were classified according to macroscopic and morphological characteristics into different actinomycete strains.

### Strain conservation

Strains were cultured on yeast malt extract agar medium composed of yeast extract (4 g/L), malt extract (10 g/L), dextrose (4 g/L), bacteriological agar (20 g/L) and pH adjusted to 7.0. The cultures on Petri dishes were incubated for 10 days at 30°C (Collections 1 and 2) or 37°C (Collection 3). Biomass or spores were then gently scraped and placed into a cryovial containing 1 mL sterile glycerol solution 80% before being stored at −80°C.

### Screening agar-plate method for PLA activity

Extracellular production of PLA activity was detected using an agar-plate method implemented in this work and based in that reported by Kouker & Jaeger (1987) with modifications. Minimal medium composed of soluble starch (1 g/L), casein (0.3 g/L), KNO_3_(2 g/L), NaCl (2 g/L), K_2_HPO_4_ (2 g/L), MgSO_4_⋅7H_2_O (0.05 g/L), CaCO_3_ (0.02 g/L), FeSO_4_⋅7H_2_O (0.01 g/L), CaCl_2_ (0.5 g/L), bacteriological agar (18 g/L), pH 7.0 was supplemented with 1% (w/v) egg yolk PC for induction and 1 mg/mL rhodamine 6G for detection of PLA activity (Okada, 2000). Substrate was previously dispersed separately with 3% (w/v) gum arabic at 10% (w/v) and rhodamine 6G was previously dissolved in ethanol and sterilized by filtration. Cultures were incubated during 10 days at conditions mentioned above. PC hydrolysis by extracellular PLA activity produced by cultures was detected by the formation of yellow fluorescent halos around actinomycete colonies visible upon UV irradiation.

### Molecular identification of microorganisms

Molecular identification was done by amplifying and sequencing a 1.3 kb fragment of the 16S rRNA gene. PCR reactions were carried out with primers 63F and 1387R (Marchesi et al., 1998) on a Veriti™ 96-well thermal cycler (Applied Biosystems, Foster City, USA) using OneTaq Hot Start DNA Polymerase (New England Biolabs). Sequencing of both strands was done at Macrogen USA Corp. Consensus sequences (Supplemental Information) were generated with the CLC Main Workbench 5.5 software package (CLCBio, Aarhus, Denmark) and compared to recorded sequences from GenBank database using the BLASTn algorithm.

### PLA activity production by SSF

Sugar-cane bagasse, polyurethane foam (PUF)/perlite (1/3 mixture), and commercial textured soy were used as SSF supports. The SSF impregnation medium for sugar-cane bagasse and PUF/perlite was composed of dextrose (5 g/L), urea (4 g/L), K_2_HPO_4_ (5 g/L), MgSO_4_ (1 g/L), CaCl_2_ (0.5 g/L) and PC inductor (40 g/L). The initial pH was adjusted to 7.0 and the medium was then finally dispersed using a mechanical homogenizer. When textured soy was used, the SSF impregnation medium consisted of glycerol (40 g/L). The agricultural residue sugar-cane bagasse and perlite were sieved to provide particles ranging between 0.8 to 1.7 mm and around 0.42 mm in size, respectively. Low density PUF was cut into 0.5 cm^3^ cubes. The three materials were washed with distilled water and then dried for two days at room temperature. Commercial textured soy received no previous treatment. SSF was performed based on the method described by Raimbault & Alazard (1980). The support was impregnated with a 1.5 times concentrated impregnation medium in the ratio of 1 g per 2 mL, respectively, and sterilized at 121°C for 15 min. Inoculum was added to obtain a final concentration of 2 ×10^7^ spores/gS and the humidity was adjusted to 75%. The inoculum was prepared by growing each strain in 250 mL Erlenmeyer flasks containing 50 mL of yeast malt extract agar medium, at 30°C (Collections 1 and 2) or 37°C (Collection 3) for 10 days. Spores were harvested with 20 mL of a solution of Tween 80 (0.01%, w/v). 125-mL Erlenmeyer flasks were packed with 20% relative volume of support, i.e., 5 g textured soy, 2.5 g sugar-cane bagasse or 0.5 g PUF with 1.5 g perlite. Culture conditions were 30 or 37°C during 10 days. Non-inoculated solid medium was incubated under the same conditions as a control.

### PLA activity extraction

Phospholipases produced in SSF cultures were extracted by adding 2.5 mM MOPS buffer (pH 7.2) containing 0.5% NLS, in a proportion of 2 mL per gram of support. The impregnated support was incubated on ice during 1 h and the liquid content was then removed gently, manually squeezed and collected. The liquid fraction was centrifuged at 13,000 rpm during 20 min to eliminate residual solids in order to obtain enzymatic extracts ready to be analyzed and were preserved on ice during treatment.

### Protein determination

Protein concentration was determined using the Bradford (1976) microassay procedure with Bio-Rad Dye Reagent. BSA was used as the reference protein and the concentration was expressed in mg/mL or µg/µL. Enzyme amount in a microplate was expressed in µg/well.

### Spectrophotometric methods for PLA activity measurements

For all enzyme activity methods applied here, one enzyme unit was defined as the amount of protein releasing 1 µmol of fatty acid per minute under assay conditions. Enzyme activity milliunits (mU) of extracts coming from SSF and determined using PC substrate, were expressed per gram of support (gS) in mU/gS. Activity measurements using EEPC substrate were also expressed in mU/gS or as specific activity as mU per mg of protein of extract in mU/mg.

#### Colorimetric HTS assay for general PLA activity detection (cHTS-PLA)

PLA activity assay was based on described methods (Mateos-Diaz et al., 2012; Camacho-Ruiz et al., 2015) with some modifications. The cHTS-PLA assay was performed using enriched egg yolk PC (≈60%, TLC) as substrate and cresol red as pH indicator to monitor the acidification induced by the release of fatty acids from PC. Reaction mixture was prepared at the moment of use with 1 volume of concentrated PC substrate solution in 2-propanol (140 mg/mL), 1 volume of 4 mM cresol red solution in 2-propanol and 18 volumes of 2.5 mM Tris buffer (pH 8.2), 150 mM NaCl, 10 mM CaCl_2_, 10 mM NaTDC, 3 mg/mL β-CD. This reaction mixture was added to a 96-wells microtiter plate (100 µL per well) using a multichannel pipette. Each sample to be tested (20 µL) at an appropriate dilution of at least 3 times in 2.5 mM Tris (pH 8.2) buffer and 20 µL of buffer or denatured sample as a blank, were then loaded in the microplate. The absorbance at 580 nm of samples against a blank without any enzyme was continuously monitored during 15 min with 30 reads at an interval of 30 s and orbital shake during 5 s before each read. Kinetic reaction was performed in a UV–Vis Microplate Spectrophotometer (xMark Bio-Rad) at 37°C. When necessary, pH of samples was adjusted to 8.2 before the assay. A standard curve established with oleic acid was linear in the 0.25 to 7 mM oleic acid final concentration, with a slope of −0.6628 and a correlation factor of 0.99. PLA activity in U/mL (units per milliliter) was calculated according to the following equation: activity (units per milliliter) = [(OD/min Reaction − OD/min Blank)/S] × (reaction volume/sample volume) × DF, where OD/min Reaction and OD/min Blank are the rates for enzyme catalyzed reaction and blank sample, respectively; S is the standard curve slope in mM per OD and DF is the dilution factor of the sample. One enzyme unit was defined as 1 µmol of fatty acid released per minute, under the assay conditions.

The PLA activity of microbial fermented solids was expressed in mU/gS through the conversion of mU/mL obtained and multiplying by the ratio of liquid used for extraction per gram of solid.

#### Ultraviolet HTS assay

PLA activity assay was based on described methods (El Alaoui et al., 2014; El Alaoui et al., 2016; Mendoza et al., 2012; Serveau-Avesque et al., 2013) with some modifications. The synthetic and specific EEPC substrate (El Alaoui et al., 2014) was first coated in 96-wells UV-microtiter plates using a 0.5 mg/mL solution prepared in ethanol and containing 0.01% BHT as an antioxidant (100 µL/well). The wells of the UV-microtiter plate were first partly dried under a fume hood and then left in a vacuum desiccator until the solvent had completely evaporated (after about 30 min). After ethanol evaporation, the EEPC-coated plates were found to be stable in the dark for at least 1 week at 4°C. PLA activity was assayed spectrophotometrically by measuring the amount of α-eleostearic acid continuously released from EEPC. The PLA activity measurement was performed with 10 mM Tris buffer (pH 8.0) containing 150 mM NaCl, 6 mM CaCl_2_, 1 mM EDTA and 3 mg/mL β-CD. The EEPC-coated wells were then washed with 0.2 mL of the assay buffer and left to equilibrate for 10 min, at 37°C with 0.2 mL of the assay buffer. The reaction was started by adding to each well, 2–20 µL of enzymatic extract previously dialyzed against 2.5 mM Tris buffer (pH 8.0) using 3 kDa cutoff membrane unit Amicon^®^ Ultra (Billerica, MA, USA), in order to eliminate pigments that interfered at 272 nm. The absorbance at 272 nm of samples against a blank without any enzyme was continuously monitored during 160 min with reads at an interval of 1 min and orbital shake during 5 s before each read. Kinetic reaction was performed in a UV–Vis Microplate Spectrophotometer (Tecan Infinite M200 Pro) at 37°C. The PLA activity was calculated from the steady-state reaction rate, expressed as the change in absorbance per minute using an apparent molar extinction coefficient of 5,320 M^−1^ cm^−1^. The PLA activity of microbial fermented solids was expressed in mU/gS through the conversion of mU/mL obtained and multiplying by the ratio of liquid used for extraction per gram of solid.

### Extraction of lipolysis products

Lipid extraction was performed immediately after the kinetic measurements from the microplate reaction medium using Folch’s method (Folch, Lees & Stanley, 1957). 20 µL of 0.1 N HCl solution were added to each microplate well to stop reaction and all the content of each microplate well was transferred into a 2 mL glass vial with a screw-cap and 500 µL of chloroform/methanol (2/1) (v/v) were added. After vigorous shaking and phase separation, the lower organic phase was collected using a Pasteur pipette and transferred to a 2 mL tube, where it was dried over anhydrous MgSO_4_. MgSO_4_ was removed by centrifugation for 1 min at 1,000 g and the clear organic phase was transferred to a 2 mL vial with a screw-cap and kept at −20°C until the analysis was performed.

### Thin-layer chromatography of lipolysis products

Residual phospholipids and lipolysis products were analyzed by thin-layer chromatography (TLC) onto 0.2 mm silica gel 60 F_254_plates using a single migration either for samples resulting from the cHTS-PLA assay or for samples resulting from the UV assay. Mobile phase used was composed by chloroform/methanol/acetic acid/NaCl 0.9% (50/25/8/4) (v/v/v/v) and 0.001% (w/v) BHT was added when EEPC was employed. Lipolysis products for samples resulting from the cHTS-PLA assay were revealed by charring the plate after spraying it with a mixture (50:50) (v/v) of saturated solution copper acetate in water and 85.5% phosphoric acid. EEPC lipolysis samples were revealed using UV light at 254 nm.

## Results

### Screening of actinomycete strains on agar-plate for extracellular PLA activity

**Figure 1:**
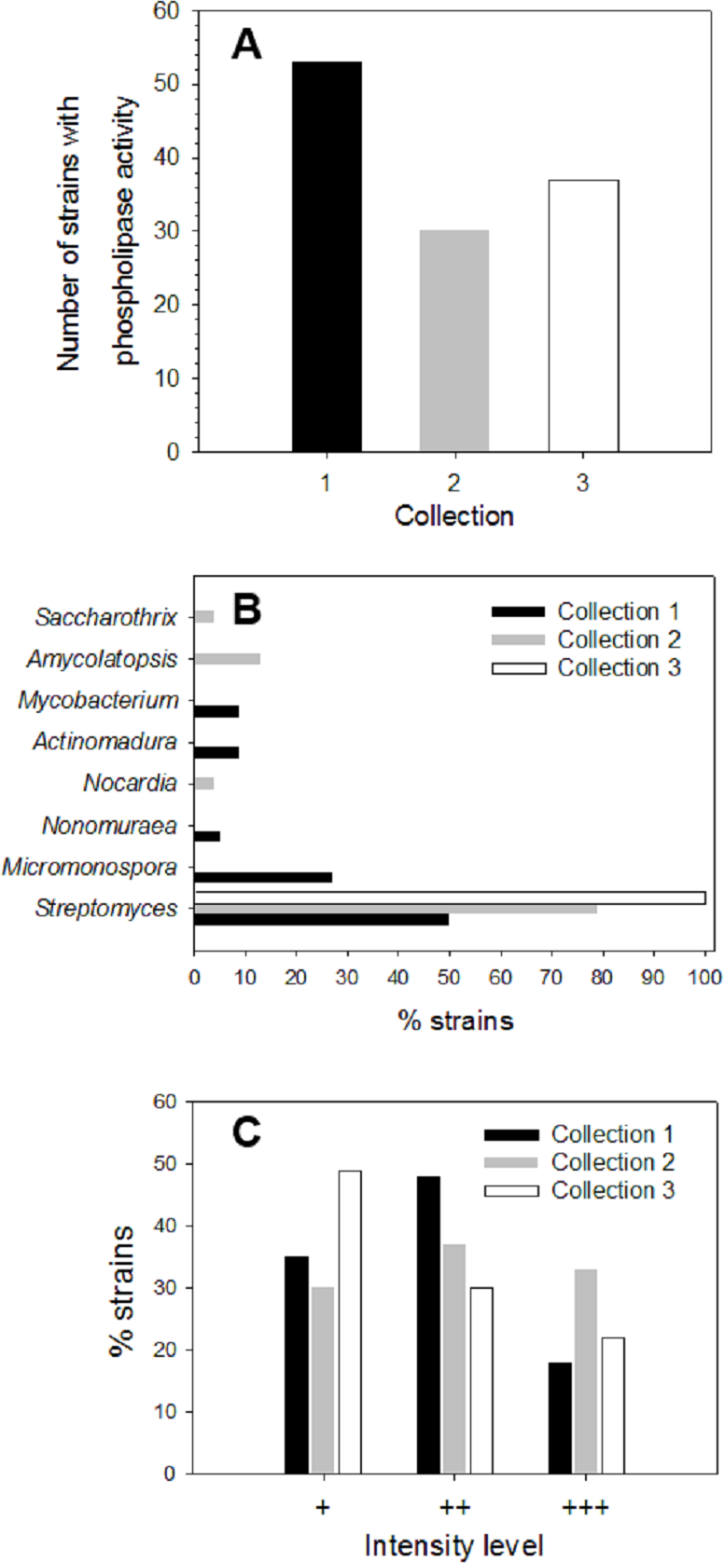
Screening of microbial collection for phospholipase activity. (A) Strains from different collections which showed extracellular phospholipase activity (primary screening) determined by using the agar-plate method with PC substrate (1%, w/v) and the fluorescent dye rhodamine 6G (1 mg/mL). (B) Genus distribution of actinomycete strains from each collection producing extracellular phospholipase activity. (C) Distribution of phospholipase activity level based in fluorescence intensities observed in the primary screening of strains from different collections. Level +: low activity, level ++: moderate activity, level +++: high activity observed in PC hydrolysis halos under UV irradiation.

About 200 strains from different soil/sediment samples from humid (Collection 1), mild (Collection 2) and extreme (Collection 3) Mexican environments were isolated by using selective microbiological culture media for actinomycetes and their ability to produce extracellular phospholipases was analyzed. Primary screening of strains to detect PLA activity was done using a sensitive agar-plate method containing the organic fluorophore rhodamine 6G and egg yolk PC substrate, as described in Materials and methods. Hydrolysis of PC substrate in plates was detected by a yellow fluorescent halo around strain colonies, visible upon UV irradiation. More than 40% of strains from each collection produced PLA activity: *n* = 53 in Collection 1 (46% of strains), *n* = 30 in Collection 2 (70% of strains), and *n* = 37 in Collection 3 (75% of strains) (Fig. 1A). According to this primary screening, 120 strains were found as important phospholipases producers. To identify the strains with PLA activity belonging to *Actinobacteria* phylum, 16S rRNA analysis was done. About 42% (*n* = 22) of strains from Collection 1, 80% (*n* = 24) of strains from Collection 2 and 22% (*n* = 8) of strains from Collection 3, were identified as actinomycetes. Genus distribution of actinomycetes for each collection is shown in Fig. 1B. In Collection 1, *Streptomyces* and *Micromonospora* were the predominant genera with about 50 and 27% of occurrence, respectively, followed by *Actinomadura* and *Mycobacterium* with a prevalence of around 9% each, and *Nonomuraea* with a lower prevalence of 5%. Results for Collection 2 showed a predominant proportion of the genus *Streptomyces* with around 79% of occurrence, followed by *Amycolaptosis* (13%), *Nocardia* and *Saccarothrix* (4% each). In Collection 3, 100% of microorganisms belonged to the genus *Streptomyces*.

During primary screening, different fluorescence intensities were observed in positive strains (Fig. S1), thus allowing an estimation of the relative levels of PLA activity in each collection (Fig. 1C).

Level 1 (+) corresponding to a low PLA activity was principally observed in Collection 3 with about 49%, followed by Collection 1 with about 35%, and finally Collection 2 with about 30% of positive strains. Moderate PLA activity represented by level 2 (+ +) was found mostly in strains from Collection 1 reaching about 48% of total strains, followed by Collection 2 with about 37% and Collection 3 with about 30%. Level 3 (+ +  +) established for high PLA activity was predominant in Collection 2 with about 33% of total strains showing an intense yellow fluorescent halo, followed by Collection 3 with about 21% and to a lesser extent, Collection 1 with 17%. Actinomycete strains that presented qualitative high PLA activity level (+ +  +) were kept for further enzyme production studies.

### Development of the colorimetric HTS assay for general PLA activity detection (cHTS-PLA)

With the aim to have a sensitive and robust method to easily quantify PLA activity and to perform the screening of a large number of samples from actinomycete strains, a continuous spectrophotometric assay referred as the cHTS-PLA assay, using microtiter plates, PC as substrate and cresol red as pH indicator was implemented based on described methods (Mateos-Diaz et al., 2012; Camacho-Ruiz et al., 2015) with some modifications. The solubility of PC (up to 100 mg/mL) was tested in several alcohols such as ethanol, 2-methyl-2-butanol, 2-methyl-2-propanol, and 2-propanol, this latter alcohol being the most suitable (Fig. S2).

**Figure 2:**
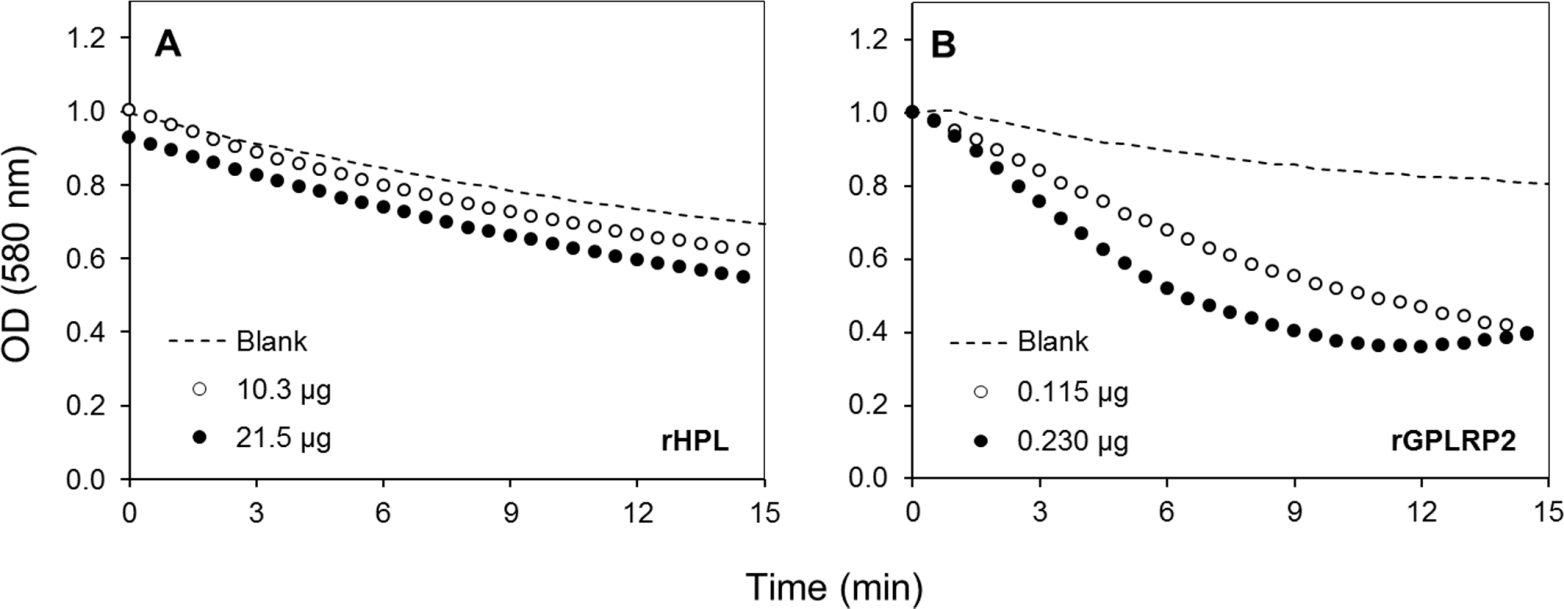
Kinetics with the cHTS-PLA assay using cresol red and PC as a substrate. (A) rHPL was used as a negative control (no phospholipase activity). (B) rGPLRP2 was used as a control enzyme with PLA_1_ activity. The decrease in OD at 580 nm was recorded for 15 min at 37 °C and the initial velocity (OD/min) was taken into account for reaction rate determination from each enzyme analyzed.

To validate the assay specificity, various purified or commercial phospholipases with *sn*-1 (PLA_1_) or *sn*-2 (PLA_2_) stereoselectivity toward phospholipids were used. tlPLA_1_ (Yang et al., 2006) and rGPLRP2 (Carriere et al., 1994; Hjorth et al., 1993) are known to display a stereopreference for the *sn*-1 position of phospholipids. hbPLA_2_ and ppPLA_2_ are known to be *sn*-2 stereoselective phospholipases (Dudler et al., 1992; Okimasu, Sasaki & Utsumi, 1984). In order to prove the specificity to detect phospholipase activity, a lipase such as the rHPL was chosen as a negative control to validate the assay. The rHPL has been reported to possess no lipolytic activity against phospholipids such as phosphatidylcholine, phosphatidylethanolamine and phosphatidylglycerol (Abousalham & Verger, 2000). Figure 2 shows the typical kinetics obtained when high amount of rHPL (21.5 µg) and low amount of rGPLRP2 (0.115 µg) were used. Significant changes in the OD decrease (ΔOD/min) were observed for rGPLRP2 in comparison with blank reaction, while with rHPL, changes in the OD with time were similar to those observed in the blank reaction and maybe attributed to a slow spontaneous hydrolysis of PC at the assay pH (8.2). The cHTS-PLA assay therefore appeared to be sensitive enough to detect PLA activity.

**Figure 3:**
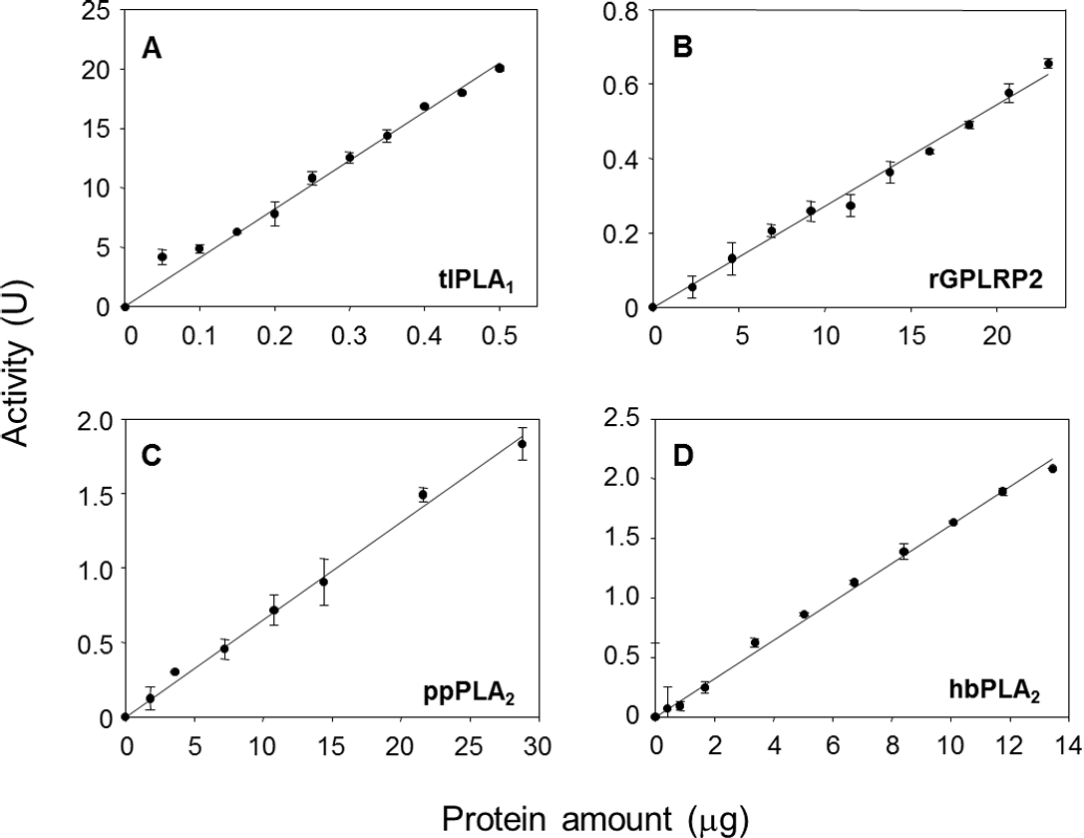
Validation of the cHTS-PLA assay by using several amounts of tlPLA_1_ (A), rGPLRP2 (B), ppPLA_2_ (C) and hbPLA_2_(D) enzymes with PC as a substrate. The decrease in OD at 580 nm was recorded for 15 min at 37 °C and the initial velocity (OD/min) was taken into account for reaction rate determination from each enzyme analyzed. Values are means ± SD (*n* = 3).

The effects of the enzyme amount on the steady-state reaction rate are shown in Fig. 3. A linear relationship was observed up to a maximum protein amount tested of 0.5 µg/well for tlPLA_1_ (*R*^2^ = 0.991, Fig. 3A), 23 µg/well for rGPLRP2 (*R*^2^ = 0.992, Fig. 3B), 28.8 µg/well for ppPLA_2_ (*R*^2^ = 0.995, Fig. 3C) and 13.4 µg/well for hbPLA_2_ (*R*^2^ = 0.997, Fig. 3D).

### Production of PLA activity by solid-state fermentation (SSF)

PLA production with actinomycete strains having high PLA activity (Level +++ in primary screening) was first attempted by submerged fermentation (SmF) using PC inductor. PLA activity from strains analyzed was however hardly detectable (Fig. S3). The actinomycete strain A119 (close to *Streptomyces djakartensis*) was then cultivated by SSF using different supports such as textured soy, sugar-cane bagasse and PUF/perlite impregnated with PC inductor. The strain A119 was able to grow in all tested supports by SSF and the PLA activity of the extracts obtained from fermented solids was measured using the developed cHTS-PLA assay with PC substrate and expressed as milliunits (mU) per gram of support (gS). The PLA activity of A119 was detected with all tested supports (Table 1); around 70 mU/gS were detected with textured soy, while with PUF/perlite, around 128 mU/gS were reached. The maximal activity value of around 240 mU/gS was obtained with sugar-cane bagasse, being about 1.8 and 3.4 times higher in comparison with PUF/perlite and textured soy, respectively. These results demonstrate for the first time, a higher production of extracellular PLA activity by actinomycete strains cultivated by SSF. Despite the fact of having done a liquid extraction from fermented solids, the PLA activity from crude enzymatic extracts obtained from these cultures was easily detectable using the implemented cHTS-PLA assay.

**Table 1:** Supports tested for extracellular phospholipase activity production (10 days at 30 °C) of actinomycete strain A119 by SSF using PC as a substrate/inductor with 75% moisture. As negative control, non-inoculated solid medium was incubated under the same conditions. Phospholipase activity of crude enzymatic extracts was measured with the cHTS-PLA assay using PC as a substrate.

SSF support	PLA activity (mU/gS)
Textured soy	69.8 ± 5.1
Sugar-cane bagasse	240.5 ± 3.7
PUF/perlite	127.7 ± 2.3

**Figure 4:**
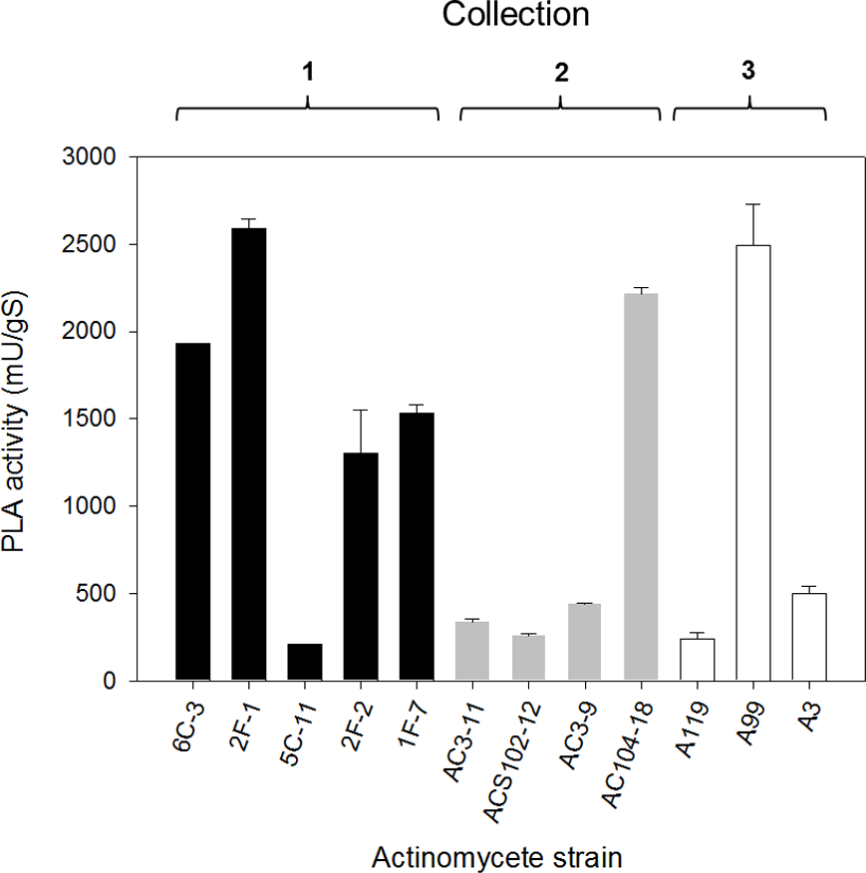
PLA activity production by solid-state fermentation with selected actinomycete strains from different collections. Sugar-cane bagasse was used as support with PC as a substrate/inductor. Extracellular PLA activity was performed using the cHTS-PLA assay with PC as a substrate. Values are means ± SD (*n* = 3).

Subsequently, once having selected the more convenient support, PLA production by selected actinomycete strains from the primary screening (level +++) was performed by SSF. The PLA activity analysis of crude extracts from SSF was made after 10 days of growth for every isolate and results are shown in Fig. 4. PLA activity of strains from Collection 1 ranging from 207 ± 4 mU/gS for 5C-11 strain to 2,591 ± 49 mU/gS for 2F-1 strain. Besides 2F-1 strain, 6C-3, 1F-7 and 2F-2 strains were also high PLA producers reaching 1,929 ± 1, 1,533 ± 49 and 1,300 ± 250 mU/gS, respectively. In Collection 2, the highest values of PLA activity were obtained for AC104-18 strain with 2,217 ± 32 mU/gS and AC3-9 strain with 440 ± 7 mU/gS; conversely, ACS102-12 strain had the lowest PLA activity value with 259 ± 11 mU/gS. In Collection 3, strains with major PLA activity were A99 with 2,495 ± 234 mU/gS, followed by A3 strain with 497 ± 46 mU/gS, and A119 with the lower value (240 ± 4 mU/gS).

**Figure 5:**
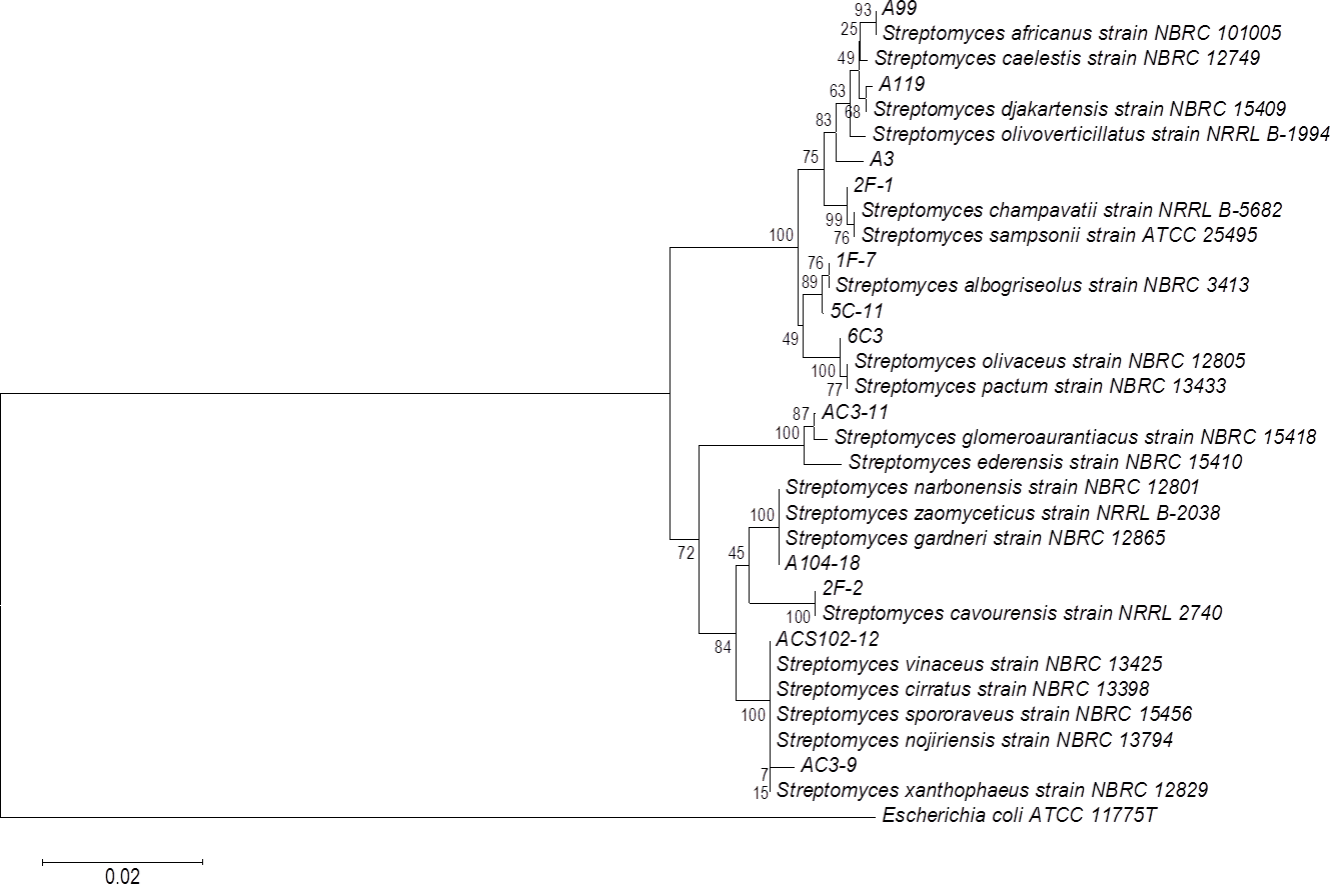
Phylogenetic tree for actinomycete strains producing PLA activity by neighbor joining analysis based on their 16S rRNA gene sequences. Numbers at the nodes are bootstrap values (of 1,000 replicates).

The phylogenetic tree of actinomycete strains producing PLA activity by SSF was obtained by neighbor joining analysis based on their 16S ribosomal RNA gene sequences (Fig. 5). There is no report of PLA activity for the actinomycete strains presented in the phylogenetic tree, which increases the chance to find novel interesting enzymes for potential applications.

**Figure 6:**
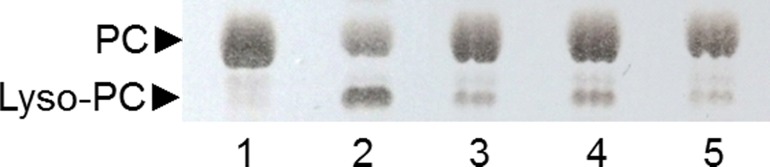
Analysis by TLC of PC hydrolysis by PLAs produced by SSF of selected strains. Lane 1: non-hydrolyzed PC substrate; Lane 2: PC substrate hydrolyzed by ppPLA_2_; lane 3: 6C-3 strain; lane 4: 2F-1 strain; lane 5: 2F-2 strain.

### Analysis of PLA activity of selected strains

**Figure 7:**
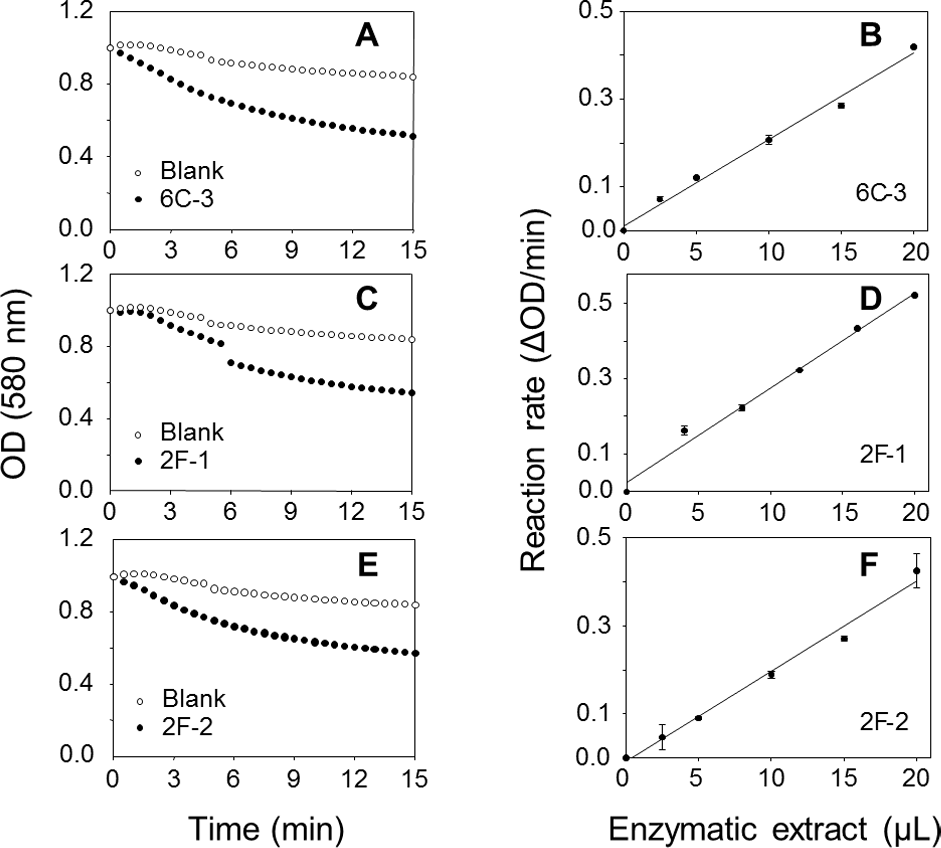
Assays of PLA activity of actinomycete strains using the cHTS-PLA assay. (A), (C), and (E) show typical kinetics of the decrease in the OD at 580 nm during the hydrolysis of PC substrate. (B), (D) and (F) show the effects of enzyme amounts on the steady-state reaction rate. The decrease in OD at 580 nm was recorded for 15 min, and the initial velocity (OD/min) was taken into account for reaction rate determination from each analyzed crude extract. The protein concentration of crude extracts of 6C-3, 2F-1 and 2F-2 cultures was 1.95, 2.82 and 2.12 mg/mL, respectively.

A TLC analysis of PC hydrolysis from the cHTS-PLA assay by phospholipases of actinomycete strains produced by SSF with sugar-cane bagasse was performed. Results showed the production of lyso-PC by the action of phospholipases A from actinomycete strains of all collections, where non accumulation or different level of accumulation of lyso-PC was observed (Fig. S4). Figure 6 displays the results obtained with 3 selected strains from Collection 1 (2F-1, close to *Streptomyces champavatii/sampsonii*; 2F-2, close to *Streptomyces caouvurensis;* and 6C-3 close to *Streptomyces olivaceus/pactum*) that stood out for the high production of lyso-PC, and thus evidence for high PLA_1_ or PLA_2_ activities in the corresponding cultures. PC hydrolysis by ppPLA_2_ was used as a positive control. PLA activity of crude extracts from 2F-1, 2F-2 and 6C-3 strains was also tested using the cHTS-PLA assay and various amounts of enzymatic extracts (Fig. 7). Typical kinetics of the decrease in the OD at 580 nm during the PC hydrolysis are shown in Figs. 7A, 7C and 7E. Variations in the steady-state reaction rate (ΔOD/min) with increasing amounts of extracts were found to be linear with time. This linear relationship was observed up to maximum protein amounts of 39 µg for 6C-3 strain (Fig. 7B, *R*^2^ = 0.988), 56 µg for 2F-1 strain (Fig. 7D, *R*^2^ = 0.985) and 42 µg for 2F-2 strain (Fig. 7F, *R*^2^ = 0.985) which was the maximum injected volume (20 µL) for each sample.

To confirm PLA production of these three actinomycete strains, a sensitive and continuous UV assay for PLA activity based on the use of the specific substrate EEPC esterified at both *sn*-1 and *sn*-2 positions with α-eleostearic acid (9*Z*, 11*E*, 13*E* octadecatrienoic acid) (El Alaoui et al., 2014) was applied. Once the EEPC substrate is coated onto a microplate well, it is stable in the presence of reaction buffer without enzyme (no increase in OD at 272 nm). When a phospholipase is injected in a well, it binds to the surface coated with EEPC and can hydrolyze it. Thanks to the presence of β-cyclodextrin, the α-eleostearic acid released from EEPC is solubilized in the aqueous phase. This leads to an increase in the OD at 272 nm that is specific to α-eleostearic acid and allows estimating PLA activity (El Alaoui et al., 2014; El Alaoui et al., 2016; Mendoza et al., 2012; Serveau-Avesque et al., 2013).

**Figure 8:**
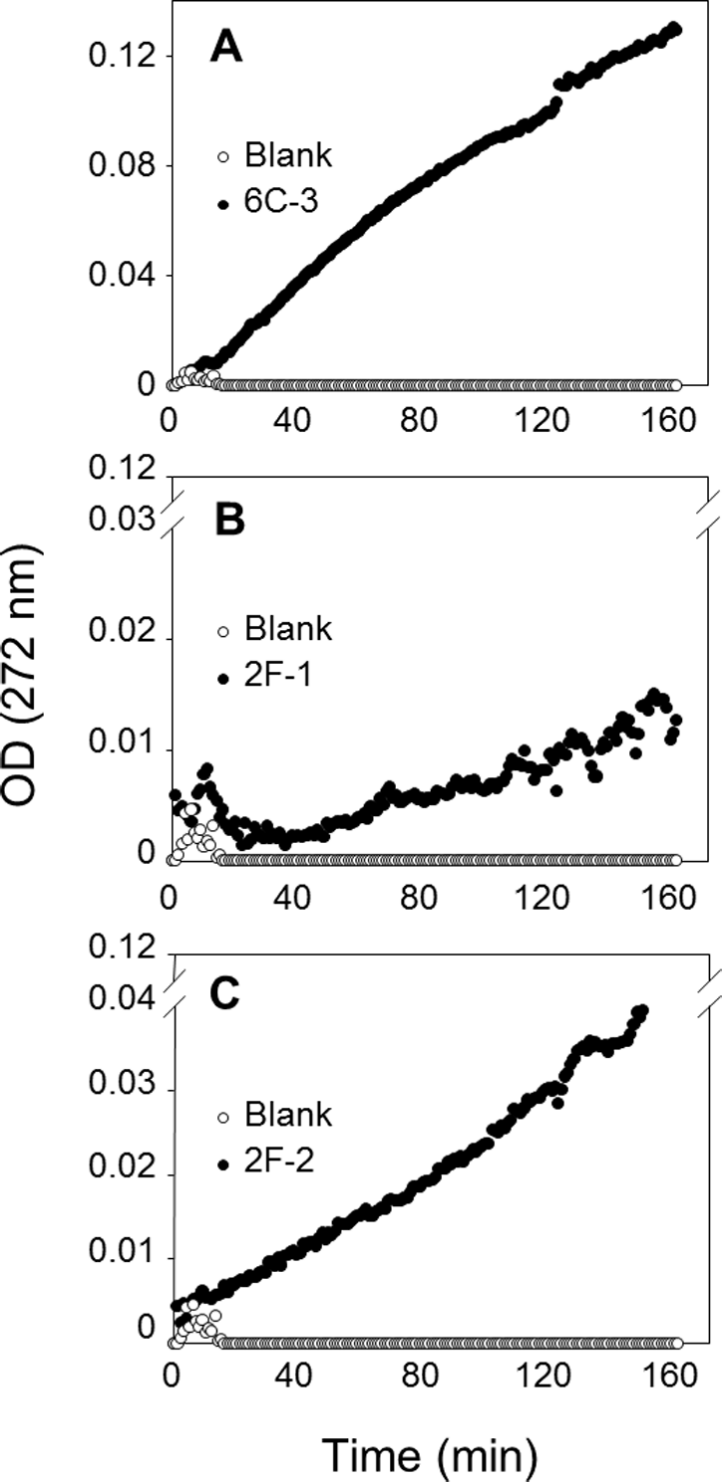
Assay of PLA activity using the synthetic and specific phospholipid EEPC coated in UV-microtiter plates. Each well contained 50 µg EEPC. Graphics show typical kinetics of the increase in OD at 272 nm during hydrolysis of EEPC substrate by 20 µL of crude enzymatic extracts injected from (A) 6C-3 (39 µg), (B) 2F-1(56.4 µg) and (C) 2F-2 (42.4 µg) strains.

The PLA activities on EEPC of extracts from 2F-1, 2F-2, and 6C-3 strains were measured using 20 µL of crude enzymatic extract (Fig. 8) and expressed as milliunits (mU) per gram of support (gS). Under these conditions, the activities of 6C-3, 2F-1 and 2F-2 were estimated to be 535, 52 and 193 mU/gS, respectively. An initial absorbance (Fig. 8B) was probably due to some detached substrate molecules that disturbed in some degree the UV measurement after sample injection into the microwell, until they finally were dispersed in the surface. This was more evident when the activity of sample was lower, and was also always observed at the beginning of blank measurements.

The reaction mixtures were recovered from the microtiter plates for lipid extraction and TLC analysis to confirm that the synthetic EEPC substrate coated in microplates was hydrolyzed by PLA present in crude enzymatic extracts from actinomycete strains (Fig. S5). The UV assay with EEPC substrate can therefore be used to confirm and measure PLA activity in crude microbial extracts.

## Discussion

### Screening of actinomycete strains on agar-plate for extracellular PLA activity

Soil samples were collected from different Mexican environments to isolate actinomycetes with the final purpose to determine their ability to produce PLA activity. The detection of phospholipases in three microbial collections was successfully achieved using a sensitive method with agar-plates containing rhodamine 6G and PC as a substrate. This method implemented in this work was effective and reliable to easily detect PC hydrolysis by extracellular phospholipases. Interestingly, in this primary screening, around 50% of strains from each collection presented this activity. Although previous works have shown determination of extracellular phospholipases using agar-plates containing either soybean lecithin or PC from fresh frozen egg yolk (Kim & Rhee, 1994; Song, Kim & Rhee, 1999), none of them included the addition of PC hydrolysis indicator. The organic fluorophore rhodamine 6G (Okada, 2000) in the agar-plate assay was added and developed here, inspired from the use of rhodamine B, another dye of the same nature, for the detection of extracellular lipase activity on agar-plates (Kouker & Jaeger, 1987). By means of the agar-plate assay containing rhodamine 6G, it was demonstrate that extracellular phospholipase activity can be detected by the PC hydrolysis producing a fluorescent halo surrounding colonies (Fig. S1) allowing the classification of strains into the three categories with low, moderate and high PLA activity (Fig. 1C). The mechanism by which rhodamine acts as an indicator of hydrolysis upon complexation with free fatty acids is not fully understood. A conceivable mechanism may be the generation of excited dimers of rhodamine which fluoresce at longer wavelengths than the excited monomer (excimer fluorescence) (Kouker & Jaeger, 1987).

### Development of the cHTS-PLA assay

With the aim to search PLA activity directly from microbial enzymatic extracts, a continuous assay was developed using microtiter plates, with commercially available enriched PC (≈60%, TLC) as substrate and cresol red as pH indicator. PLA activity measurement was based on the decrease of pH indicator optical density due to protonation caused by the release of free fatty acids during the hydrolysis of substrate and thus acidification of the reaction media (Camacho-Ruiz et al., 2015). A lipase (rHPL) and enzymes with PLA activity were used to validate the method. As expected, rHPL was not able to hydrolyze the PC (Fig. 2), while in the case of enzymes with PLA activity, the rate of lipolysis was found to be linear with time and proportional to the amount of enzyme unto the maximum amount added in each case (Fig. 3). Since each of these enzymes has a different specific activity toward the PC substrate, the range of enzyme amount to have a proportional velocity was different between them. A similar HTS assay has been reported by Mateos-Diaz et al. (2012) to determine esterase/lipase activity using tributyrin or trioctanoin substrate and *p*-nitrophenol as pH indicator at pH 7.2. They applied it to determine lipase activity in crude extracts from fungal strains *Rhizopus* sp.8a and *Aspergillus ochraceus*. Although previous PLA activity assays using phospholipid substrate combined or not with chromogenic molecules have been proposed (Lobo de Araujo & Radvanyi, 1987; Petrovic et al., 2001; Price, 2007), they were not designed to be used with crude microbial extracts and some of them were not compatible with HTS. Hence, it was showed a clear evidence that the continuous cHTS-PLA assay using PC substrate and cresol red at pH 8.2 developed in this work is a convenient tool for high-throughput screening.

### PLA activity in actinomycetes

Analysis of 16S rRNA sequences provided enough information to identify actinomycete strains up to the genus level. Those from the genus *Streptomyces* were found as notable sources of phospholipase activity. Our findings (Figs. 4 and 5) are in agreement with some literature reports indicating the presence of bacterial phospholipase activity in several *Streptomyces* species based on multiple sequence alignment (Yang & Roberts, 2002; McMahon et al., 2012). In particular, a secreted PLA_2_ produced by *Streptomyces violaceoruber* A-2688 isolated from a soil sample, was described as the first PLA_2_ identified in prokaryote (Sugiyama et al., 2002; Takemori et al., 2012). Similarly, a PLA_1_ and a phospholipase B have also been reported in the *Streptomyces* genus (Sugimori, Kano & Matsumoto, 2012; Matsumoto et al., 2013). Some authors have also reported the presence of lipases in actinomycete strains (Sztajer, Maliszewska & Wieczorek, 1988) while others did not observe the production of lipolytic enzymes (Basha et al., 2009).

### Production of PLA by solid-state fermentation

PLA production was initially done using submerged fermentation cultures, however, low production yields were obtained. To deal with this problem, the SSF was proposed as an alternative since several studies have stressed the advantages of SSF over SmF, attributed to the higher biomass growth rates and the lower enzyme proteolysis showing that SSF gives higher enzyme yields (Aguilar et al., 2001; Diaz-Godinez et al., 2001; Romero-Gomez, Augur & Viniegra-Gonzalez, 2000). In order to achieve higher PLA production yields, SSF was performed using three different supports and PC inductor contained into the impregnation media. The A119 strain (close to *Streptomyces djakartensis*) cultured on three different supports produced PLA activity in all of them. SSF cultures on sugar-cane bagasse support showed the highest yield reaching 240 mU/gS compared with 128 mU/gS and 70 mU/gS produced on PUF/perlite and textured soy supports, respectively (Table 1). Based on these findings, sugar-cane bagasse was selected as a convenient support for actinomycete SSF cultures for PLA production. Our results are in line with publications proposing SSF as a potent tool for enzyme production and an alternative to SmF (Raimbault & Alazard, 1980; Aguilar et al., 2001; Hansen et al., 2015; Pandey et al., 1999; Thomas, Larroche & Pandey, 2013; Viniegra-Gonzalez et al., 2003). The direct comparison of SSF and SmF using various parameters such as growth rate, productivity or volume activity for the production of enzymes is in favor of SSF in most cases (Holker, Hofer & Lenz, 2004). It has been reported that SSF system achieved higher productivity for some enzymes mainly because of three factors: higher levels of biomass production, apparent resistance to catabolite repression and reduced breakdown of enzymes by proteases (Viniegra-Gonzalez et al., 2003). The higher production of enzymes in SSF may be due to the fact that it takes place in the absence or near absence of free water, thus being close to the natural environment to which microorganisms are adapted (Pandey, Soccol & Mitchell, 2000). Moreover, many reports of SSF have been recently published in which emphasis is given to the application of agricultural by-products for the production of fine chemicals and lipolytic enzymes (Treichel et al., 2010). Utilization of agro-industrial wastes provides alternative substrates and may help solving pollution problems, which otherwise might be caused by their disposal (Treichel et al., 2010). Particularly, the use of sugar-cane bagasse has been proposed to produce interesting molecules (Lakshminarayana et al., 1975) and was also applied for lipase production (Dutra et al., 2008; Mateos-Diaz et al., 2006; Rodriguez et al., 2006). Phospholipase production by actinomycetes using SSF using sugar-cane bagasse is however reported here for the first time (Fig. 4).

Production of PLA activity by bacteria have been only reported using SmF and different protocols for enzyme quantification have been used. In that regard, a native membrane-bound PLA_1_ purified from *Escherichia coli* (Scandella & Kornberg, 1971) showed 0.0084 U/mL of phospholipase activity in the crude extract determined using the radioactive substrate [^32^P]phosphatidylglycerol. A native PLA_1_ from *Serratia* sp. (Kim & Rhee, 1996) produced around 1.2 U/mL of phospholipase activity determined using the pH titration method at 50 °C with egg yolk lecithin as a substrate. Similarly, a native PLA_1_ from *Streptomyces albidoflavus* produced 1.5 U/mL of phospholipase activity in the culture supernatant (Sugimori, Kano & Matsumoto, 2012) determined using a fluorometric method with a BODIPY(R) fluorescent dye-labeled substrate. In other hand, a microbial PLA_1_ was isolated from a mutant strain of the ciliated protozoan *Tetrahymena thermophile* and had 0.068 U/mL of phospholipase activity in the crude extract (Guberman et al., 1999) determined with the radioactive substrate 1-palmitoyl-2-[1-^14^C]linoleoylphosphatidylcholine. Despite the differences in activity quantification, comparison of these values with the PLA activities obtained here with the cHTS-PLA assay using PC as a substrate (2.59 U/gS (3.45 U/mL) for 2F-1, 1.93 U/gS (2.57 U/mL) for 6C-3 and 1.53 U/gS (2.04 U/mL) for 2F-2 strain), clearly demonstrates that their production by SSF with sugar-cane bagasse at the conditions employed here, appears to improve profiles for native microbial PLA production.

By means of the TLC analysis of PC hydrolysis from the cHTS-PLA assay, it was demonstrate the presence of PLAs in the crude extracts of actinomycete strains produced by SSF with sugar-cane bagasse as support. Moreover, it was shown that the synthetic and specific EEPC substrate coated into microtiter plates can be used to confirm the presence of PLA in crude microbial extracts (Fig. 8). As proposed previously (Mendoza et al., 2012; Pencreac’h et al., 2002; Serveau-Avesque et al., 2013), synthetic substrates containing α-eleostearic acid appears to be stable, sensitive and convenient tools for measuring the activity and specificity of lipolytic enzymes using UV detection of the released fatty acids, either on TLC plates or microtiter plates. Finally, the value of activity detected with EEPC assay was smaller and different to activity detected with the cHTS-PLA assay for the three selected microbial extracts. It was found that the PLA activity detected with cHTS-PLA assay on PC substrate was about 3.6, 49 and 8 times more compared with the PLA activity on EEPC for 6C-3, 2F-1 and 2F-2 strains extracts, respectively. These kinds of differences has been observed for example in the PLA activity determination of rGPLRP2 on C8-PC (1,2-dioctanoyl phosphatidylcholine) and egg PC with values of 551 ± 50 and 812 ± 100 U/mg, respectively, using the pH-stat technique in which the conditions assay consisted of a mechanically stirred emulsion of substrate previously emulsified by ultrasonic treatment (Dridi et al., 2013). In other hand, the PLA activity of rGPLRP2 on coated EOPC (1-α-eleostearoyl-2-octadecyl-*rac*-glycero-3-phosphocholine) was reported with a value of 0.3 ± 0.02 U/mg (El Alaoui et al., 2016), while the PLA activity of rGPLRP2 with the cHTS-PLA assay developed in this work was of 25.2 ± 0.9, about 84 times more compared with EOPC, and 32 times less compared to pH-stat technique using the same substrate.

These differences can be attributed to the specificity of each enzyme for each kind of substrate, the presentation of substrate (e.g., coated monocapes for EEPC and an emulsion for PC substrate) or the conditions of the assay (e.g., mechanical stirring, orbital shaking).

## Conclusion

Novel microbial PLAs from actinomycetes mostly belonging to *Streptomyces* and *Micromonspora* genus were identified in this work, by means of a set of efficient implemented methods that pave the way to HTS of PLA activity in crude microbial enzymatic extracts at a larger scale. The cHTS-PLA assay developed here is convenient and rapid for easy detection of PLA activity in crude microbial enzymatic extracts and can be also proposed as a routine assay for PLA activity determination during enzyme purification, directed evolution or mutagenesis approaches. In addition, the production of PLA activity by actinomycetes using SSF with the agricultural by-product sugar-cane bagasse was developed. This new process will allow the production of novel PLA with potential applications in biotechnology.

##  Supplemental Information

10.7717/peerj.3524/supp-1 Figure S1 Detection of phospholipase activity using the agar-plate method with enriched media containing egg yolk PC and rhodamine 6G as indicator exposed at UV light (254 nm) *Pichia pastoris* strains cultivated after 4 days at 30 °C in which (A) *P. pastoris* wild-type strain (X-33) without gene insertion, and (B) *P. pastoris* expressing the enzyme GPLRP2 which has been reported with PLA activity. Actinomycete strains cultivated during 10 days at 30 °C in which (C) 7E-1 strain (Collection 1) without PLA activity production, and (D) 6C-3 strain (Collection 1) with PLA activity production. The yellow fluorescent halo around the colony indicates the PLA activity production.

10.7717/peerj.3524/supp-2 Figure S2 Solubility test of egg PC (≈ 60%) with several alcohols (A): egg PC alone; (B): egg PC + ethanol; (C): egg PC + 2-methyl-2-butanol; (D): egg PC +2-methyl-2-propanol; (E): egg PC + 2-propanol. Alcohols were added to egg PC and mixed by vortex during 1.5 min at 25 °C to have a final concentration of 120 mg/mL. In B, egg PC had better solubility but a small white precipitate was observed; in C and D, an abundant amber precipitate was observed; in E, egg PC was solubilized and no precipitate was observed.

10.7717/peerj.3524/supp-3 Figure S3 Kinetics of activity obtained for A99 strain extracts produced by liquid (SmF) and solid state fermentation (SSF) after 10 days at 30 °C Samples were diluted 2 and 5 fold for SmF and SFF extracts, respectively; 20 uL of diluted sample were added to hydrolysis reaction using PC as a substrate through the cHTS-PLA method.

10.7717/peerj.3524/supp-4 Figure S4 Analysis by TLC of PC hydrolysis by PLAs produced by SSF of selected strains of each Collection Lane 1: non-hydrolyzed PC substrate; Lane 2: PC hydrolyzed by A99 strain extract (Collection 3); Lane 3: PC hydrolyzed by AC3-11 strain extract (Collection 2); Lane 4: PC hydrolyzed by 6C-3 strain extract (Collection 1). The accumulation of Lyso-PC was the evidence of PLA activity.

10.7717/peerj.3524/supp-5 Figure S5 Analysis by TLC of EEPC hydrolysis revealed through UV light (254 nm) Lane 1, non hydrolyzed EEPC; lane 2, EEPC hydrolyzed by ppPLA_2; lane 3, EEPC hydrolyzed by *6C-3 strain* crude extract produced by SSF; lane 4, purified a-eleostearic acid. The mobile phase employed was chloroform/methanol/acetic acid/NaCl 150 mM, 50:25:8:4 (v/v/v/v) containing 0.001% (w/v) BHT.

10.7717/peerj.3524/supp-6 Supplemental Information 1 Raw data of 16S consensus sequences

## Abbreviations

β-CD: β-cyclodextrin
BHT: butylhydroxytoluene
BSA: bovine serum albumin
C8-PC: (1,2-dioctanoyl phosphatidylcholine)
CHAPS: 3-[(3-cholamidopropyl)dimethylammonio]-1-propanesulfonate
cHTS-PLA: colorimetric HTS assay for general PLA activity detection
EDTA: ethylenediaminetetraacetic acid
EEPC: 1,2-α-eleostearoyl-*sn*-glycero-3-phosphocholine
EOPC: (1-α-eleostearoyl-2-octadecyl-*rac*-glycero-3-phosphocholine)
FFA: free fatty acid
gS: grams of support
hbPLA_2_: honey bee venom PLA_2_
HTS: high-throughput screening
MOPS: 3-(N-morpholino)propanesulfonic acid
mU: enzyme activity miliUnit
NaTDC: sodium taurodeoxycholate
NLS: N-lauroylsarcosine
PC: phosphatidylcholine
PHIBLA: pH indicator-based lipase assay
PLA: phospholipase A
PLA_1_: phospholipase A_1_
PLA_2_: phospholipase A_2_
ppPLA_2_: porcine pancreatic PLA_2_
PUF: polyurethane foam
SmF: submerged fermentation
SSF: solid-state fermentation
rGPLRP2: recombinant guinea pig pancreatic lipase-related protein 2
rHPL: recombinant human pancreatic lipase
Tris: 2-amino-2-(hydroxymethyl)-1,3-propanediol
TLC: thin-layer chromatography
tlPLA_1_: PLA_1_ from *Thermomyces lanuginosus*
TAG: triacylglycerol
U: enzyme activity unit
UV: ultraviolet

## Additional Information and Declarations

### Data Availability

The following information was supplied regarding data availability: The raw data has been supplied as a Supplementary File.
