# Correction: Screening of phospholipase A activity and its production by new actinomycete strains cultivated by solid-state fermentation

**DOI:** 10.7717/peerj.3524/correction-1

**Published:** 2017-08-11

**Authors:** Priscila Sutto-Ortiz, María de los Angeles Camacho-Ruiz, Manuel R. Kirchmayr, Meddy El Alaoui, Rosa María Camacho-Ruiz, Juan Carlos Mateos-Díaz, Alexandre Noiriel, Frédéric Carrière, Abdelkarim Abousalham, Jorge A. Rodríguez

**Affiliations:** 1 Biotecnología Industrial, Centro de Investigación y Asistencia en Tecnología y Diseño del Estado de Jalisco A.C., Zapopan, Jalisco, Mexico; 2 Univ Lyon, Université Lyon 1, Institut de Chimie et de Biochimie Moléculaires et Supramoléculaires (ICBMS), UMR 5246, Métabolisme, Enzymes et Mécanismes Moléculaires (MEM2), Villeurbanne Cedex, France; 3 CNRS, Aix Marseille Université, UMR 7282, Enzymologie Interfaciale et de Physiologie de la Lipolyse, Marseille, France; 4 Departamento de Fundamentos del Conocimiento, Centro Universitario del Norte, Universidad de Guadalajara, Colotlán, Jalisco, Mexico

The author list should have included Meddy El Alaoui as the 4th author in the author list. Meddy El Alaoui prepared the phospholipid substrate EEPC used to assay PLA activity, contributed to reagents/materials/analysis tools, and reviewed drafts of the paper. They were mistakenly omitted from the author list during the review process due to an involuntary omission.

