# Supplementary figures and images for: Screening of phospholipase A activity and its production by new actinomycete strains cultivated by solid-state fermentation
Source: PeerJ. 2017 Jul 6;5:e3524. doi: 10.7717/peerj.3524 (PMC5501967; doi:10.7717/peerj.3524)

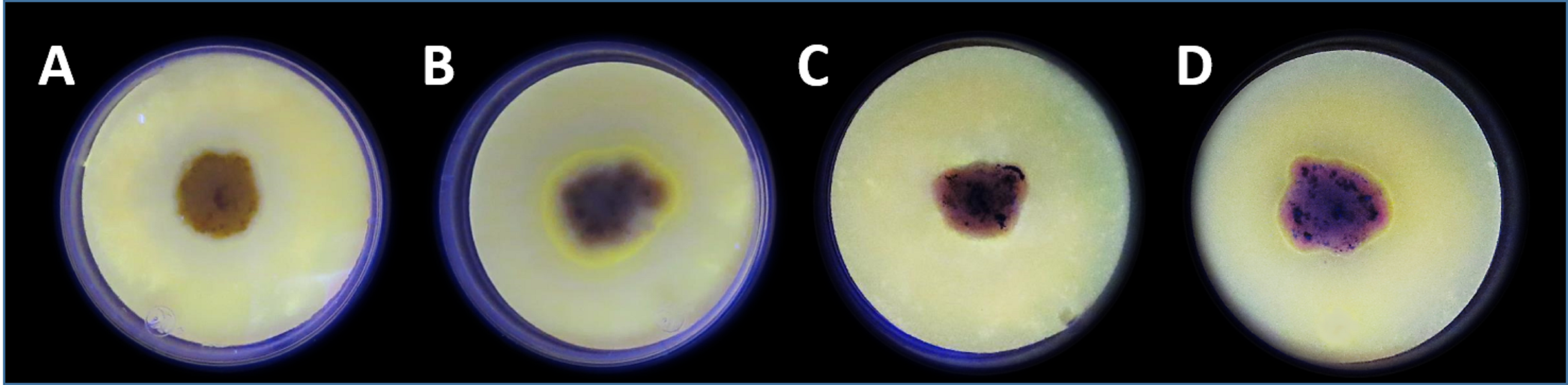

Supplement: Figure S1 — Pichia pastoris strains cultivated after 4 days at 30 °C in which (A) P. pastoris wild-type strain (X-33) without gene insertion, and (B) P. pastoris expressing the enzyme GPLRP2 which has been reported with PLA activity. Actinomycete strains cultivated during 10 days at 30 °C in which (C) 7E-1 strain (Collection 1) without PLA activity production, and (D) 6C-3 strain (Collection 1) with PLA activity production. The yellow fluorescent halo around the colony indicates the PLA activity production. [file peerj-05-3524-s001.png]

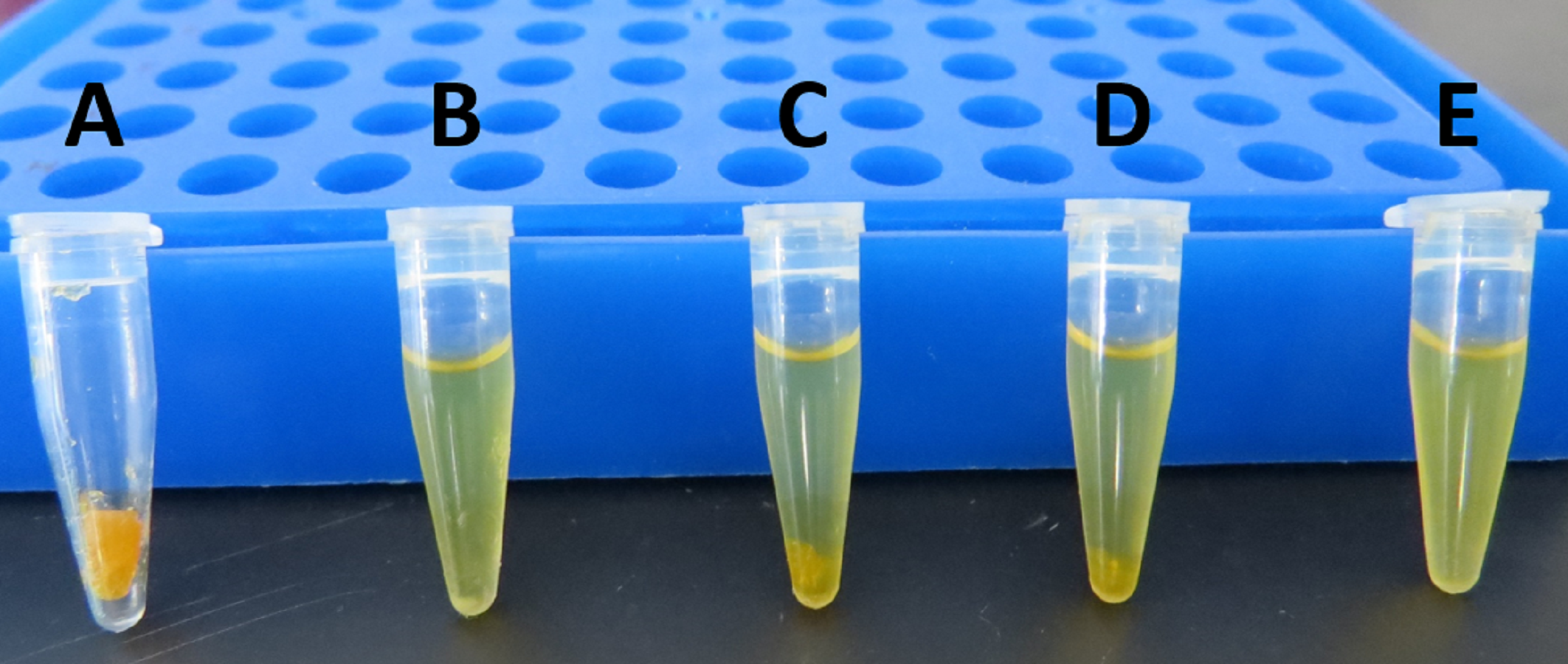

Supplement: Figure S2 — (A): egg PC alone; (B): egg PC + ethanol; (C): egg PC + 2-methyl-2-butanol; (D): egg PC +2-methyl-2-propanol; (E): egg PC + 2-propanol. Alcohols were added to egg PC and mixed by vortex during 1.5 min at 25 °C to have a final concentration of 120 mg/mL. In B, egg PC had better solubility but a small white precipitate was observed; in C and D, an abundant amber precipitate was observed; in E, egg PC was solubilized and no precipitate was observed. [file peerj-05-3524-s002.png]

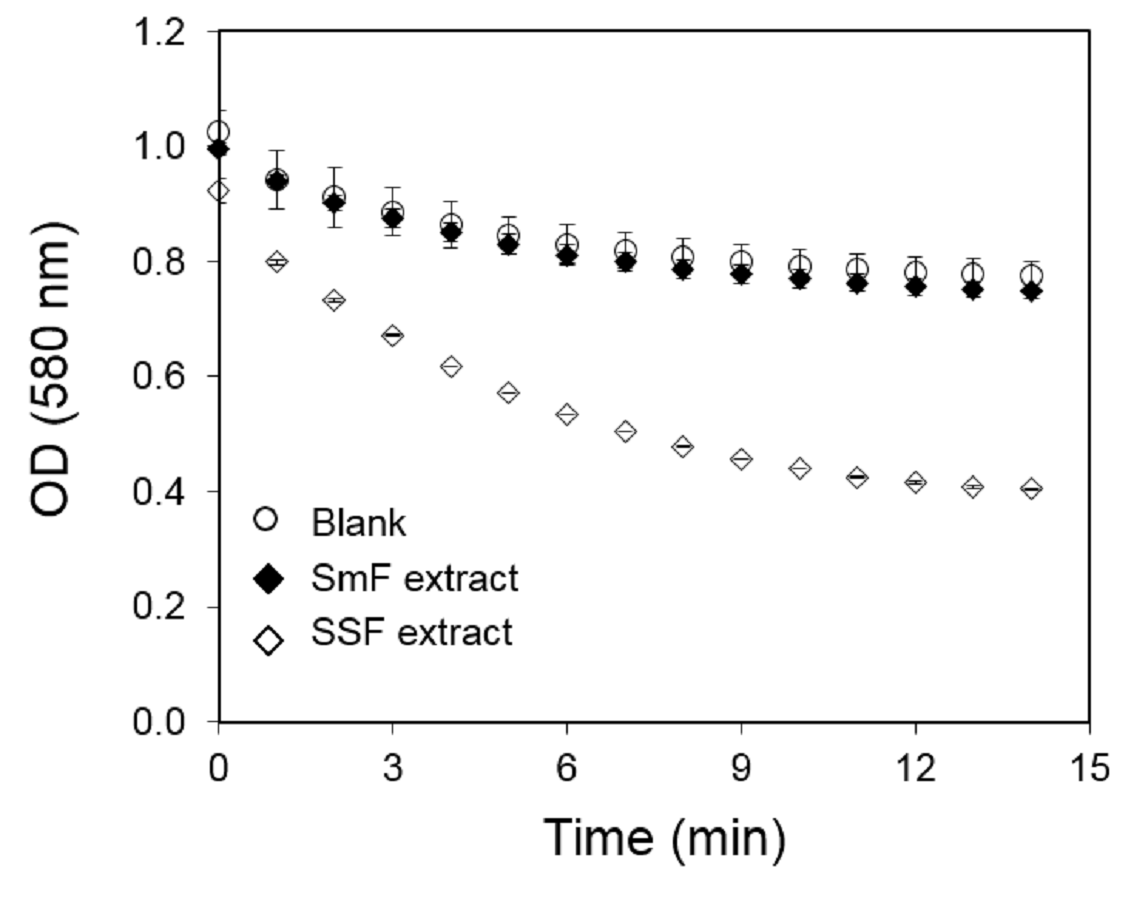

Supplement: Figure S3 — Samples were diluted 2 and 5 fold for SmF and SFF extracts, respectively; 20 uL of diluted sample were added to hydrolysis reaction using PC as a substrate through the cHTS-PLA method. [file peerj-05-3524-s003.png]

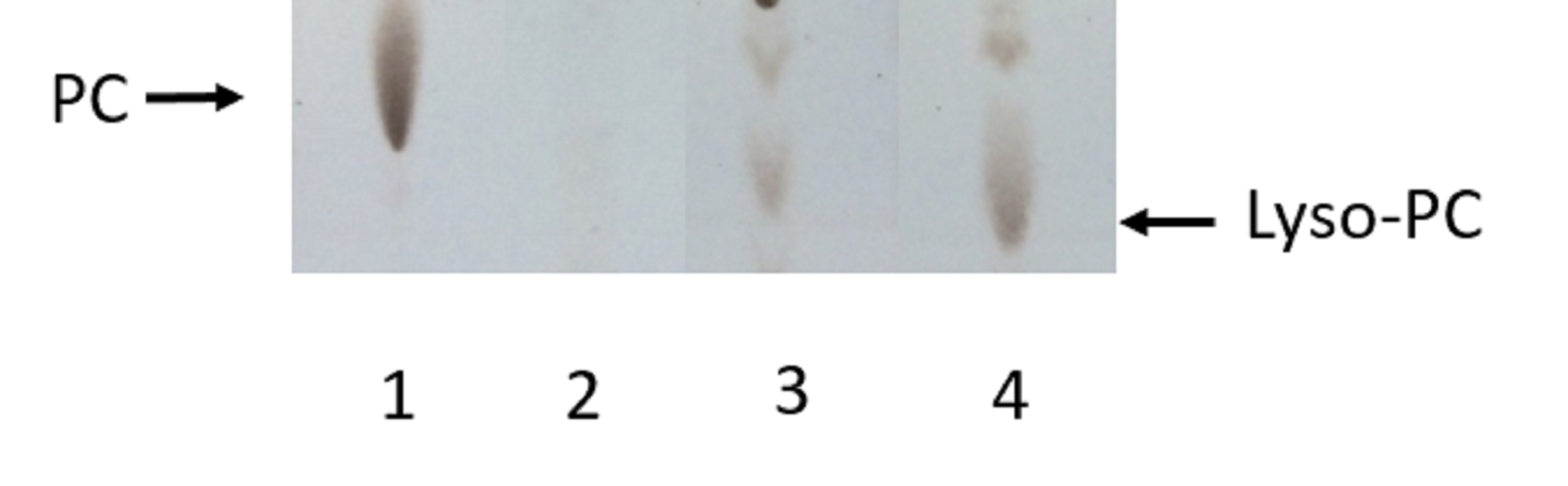

Supplement: Figure S4 — Lane 1: non-hydrolyzed PC substrate; Lane 2: PC hydrolyzed by A99 strain extract (Collection 3); Lane 3: PC hydrolyzed by AC3-11 strain extract (Collection 2); Lane 4: PC hydrolyzed by 6C-3 strain extract (Collection 1). The accumulation of Lyso-PC was the evidence of PLA activity. [file peerj-05-3524-s004.png]

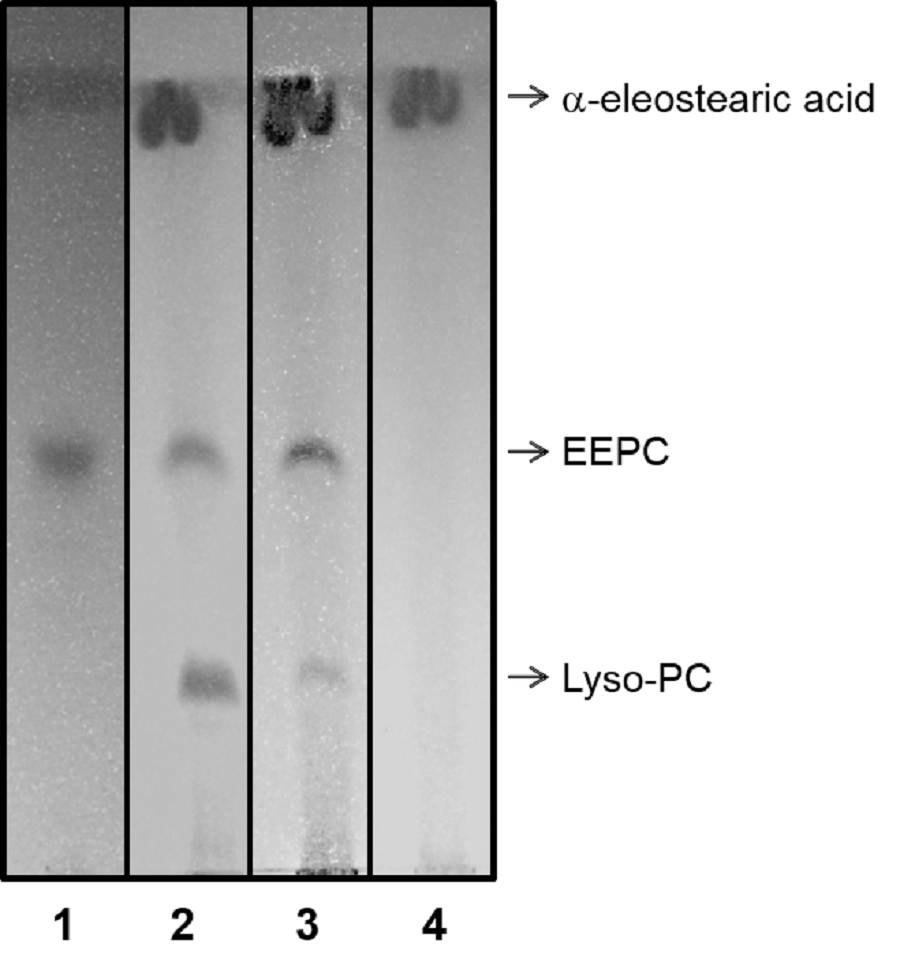

Supplement: Figure S5 — Lane 1, non hydrolyzed EEPC; lane 2, EEPC hydrolyzed by ppPLA_2; lane 3, EEPC hydrolyzed by 6C-3 strain crude extract produced by SSF; lane 4, purified a-eleostearic acid. The mobile phase employed was chloroform/methanol/acetic acid/NaCl 150 mM, 50:25:8:4 (v/v/v/v) containing 0.001% (w/v) BHT. [file peerj-05-3524-s005.png]
